# Heterogeneity in polyamine metabolism dictates prognosis and immune checkpoint blockade response in hepatocellular carcinoma

**DOI:** 10.3389/fimmu.2025.1516332

**Published:** 2025-02-06

**Authors:** Jianyan Pan, Zhong Lin, Qinchun Pan, Tao Zhu

**Affiliations:** ^1^ Department of Birth Health and Genetics, The Reproductive Hospital of Guangxi Zhuang Autonomous Region, Nanning, China; ^2^ School of Medicine & Health, Guangxi Vocational & Technical Institute of Industry, Nanning, China; ^3^ Department of Pharmacy, The Second Affiliated Hospital, Zhejiang University School of Medicine, Hangzhou, China

**Keywords:** hepatocellular carcinoma, polyamine, metabolic heterogeneity, immunotherapy, prognosis

## Abstract

Immune checkpoint blockade holds promise in hepatocellular carcinoma (HCC) treatment, but its efficacy remains limited. Dysregulated polyamine metabolism and its interaction with oncogenic pathways promote tumor progression. However, the heterogeneity of polyamine metabolism and its effects on the immune microenvironment and response to immunotherapy in HCC remain unclear. Here, we aimed to investigate the prognostic and immunotherapeutic implications of polyamine metabolism in HCC. Based on polyamine-related genes, HCC patients were categorized into two clusters with distinct survival outcomes. We developed a polyamine-related signature, termed PAscore, which was found to be a strong predictor of both poor prognosis and reduced immunocyte infiltration. Notably, a high PAscore was also associated with decreased sensitivity to immunotherapy. Within the HCC microenvironment, malignant cells exhibited polyamine metabolic heterogeneity, those with high polyamine metabolic activity showed altered hallmark pathway signatures and increased communication with myeloid cells. *In vitro* experiments suggested that FIRRE, the gene with the greatest impact on the PAscore, significantly contributed to HCC proliferation and metastasis. This study underscores the potential of our polyamine-related signature in predicting the prognosis and immunotherapy response in HCC patients, and also reveals the polyamine metabolic heterogeneity among HCC cells that influences their crosstalk with infiltrating myeloid cells.

## 1 Introduction

Primary liver cancer is one of the most prevalent malignant tumors in the digestive system, ranking sixth in incidence among all malignancies. It has the third highest mortality rate, following lung and colorectal cancers, and this rate has increased over the past decade (1, 2). It includes hepatocellular carcinoma (HCC), intrahepatic cholangiocarcinoma (ICC), and mixed hepatocellular-cholangiocarcinoma. HCC, which originates from liver cells, is the most prevalent histological type, accounting for approximately 90% of all primary cases (3). The primary treatment strategies for liver cancer include surgical resection, interventional therapy, radiotherapy, and chemotherapy. In recent years, immunotherapy that blocks PD-1/PD-L1 and CTLA-4 signals have emerged as a promising approach that can bring survival benefits for HCC patients, which makes it a key area of research (4, 5). However, despite these treatment options, the overall therapeutic outcomes remain limited, with a five-year survival rate of only about 20%. The challenges in treating HCC are primarily due to tumor heterogeneity, low drug response rates or resistance, and the limited availability of effective drugs targeting key driver mutations (6–8). There is an urgent need for reliable predictive models to identify high-risk HCC patients for timely and effective treatment.

The polyamines (putrescine, spermidine and spermine) present in mammalian cells are essential for cell function and growth. They are involved in many cellular activities, such as chromatin structuring, gene regulation, protein and nucleic acid synthesis, cell differentiation and apoptosis, and intercellular communication (9). Metabolic reprogramming is a hallmark of cancer, and dysregulated polyamine metabolism is prevalent in many cancer types. The rate-limiting enzymes of polyamine biosynthesis, ODC and AMD1, are direct transcriptional targets of the oncogene MYC (10, 11). The upregulation of MYC expression, which occurs in nearly all tumor types due to gene mutations, is closely associated with increased polyamine biosynthesis (9, 12). Aberrant polyamine metabolism, whether driving or resulting from oncogenic pathways, impacts cancer cell survival, contributes to acquired drug resistance, and alters the tumor microenvironment (13, 14). Targeting tumor polyamine metabolism is therefore considered a rational strategy for therapeutic intervention. In HCC, Liu et al. proposed that polyamines regulate mitochondrial metabolism to influence the differentiation of macrophages and T cells, thereby promoting the formation of an immunosuppressive microenvironment (15). To date, whether HCC exhibits heterogeneity in polyamine metabolism and the prognostic significance of polyamine metabolism remain to be elucidated.

In this study, single-cell RNA sequencing data of HCC were utilized to examine polyamine metabolism activity at the single-cell level, and bulk transcriptome sequencing data were used to explore the expression patterns of polyamine-related genes in HCC. Polyamine-related genes correlated with the overall survival of HCC patients were identified, and a polyamine metabolism-related prognostic model, termed PAscore, was established to predict the tumor microenvironment, clinical outcomes, and responses to therapies in HCC. This study presents a prognostic signature based on polyamine homeostasis and highlights the potential of targeting polyamine metabolism as a therapeutic strategy for HCC patients.

## 2 Materials and methods

### 2.1 Bulk RNA−seq and clinical data acquisition

Transcriptomic data in counts and TPM formats, somatic mutation data in the mutation annotation format, and associated clinical information for HCC were retrieved from The Cancer Genome Atlas (TCGA) using the R package ‘TCGAbiolinks’ (16). This cohort includes 374 tumor samples and 50 normal samples. Samples with overall survival less than 30 days or lacking complete survival data were excluded. Gene expression data and clinical information from an independent HCC cohort, GSE14520, were downloaded from the Gene Expression Omnibus (GEO) database to serve as a validation set. This cohort includes 225 tumor samples and 220 normal samples. Similarly, only patients with corresponding survival data and survival greater than 30 days were included in the analysis. For duplicated gene symbols, the gene with the highest average expression value was retained. The LICA-FR dataset containing 160 HCC samples was downloaded from the International Cancer Genome Consortium (ICGC). A curated list of polyamine-related genes was derived from the GeneCards database using a relevance score threshold greater than 1 (**Supplementary Table 1**).

### 2.2 Single−cell RNA sequencing analysis

The GSE166635 single-cell transcriptome data for HCC were collected from Tumor Immune Single-cell Hub (TISCH) database. Data processing was conducted using the ‘Seurat’ R package, following the procedures described in the package tutorial. Briefly, cells with < 500 or > 7,500 expressed genes, as well as those with mitochondrial gene expression > 15%, were excluded. After cell quality control, the raw counts were log-normalized and scaled, and principal component analysis (PCA) was performed using highly variable genes. Cell clustering was performed by selecting the first 15 components and employing the Louvain algorithm, followed by dimensionality reduction and visualization using unified manifold approximation and projection (UMAP). We then annotated each cell cluster using the annotations provided with the downloaded dataset. For the sub-clustering of malignant cells, the raw data of malignant cells were extracted and the procedures mentioned above were repeated.

### 2.3 GSVA analysis of pathway activity and cell–cell communication analysis

For pathway activity analysis, the ‘GSVA’ R package was employed to evaluate the enrichment of relevant gene sets using normalized gene expression data from each sub-cluster of malignant cells. The gene sets used for Gene Set Variation Analysis (GSVA), including the reactome metabolism of polyamines gene set and the hallmark gene sets, were retrieved from the Molecular Signatures Database (MSigDB, https://www.gsea-msigdb.org/gsea/msigdb). Cell-cell communications were analyzed using the ‘CellChat’ package, with the number of ligand-receptor pairs and communication intensity evaluated based on normalized gene expression data.

### 2.4 Identification of differentially expressed genes and prognostic genes

The counts format of the bulk RNA-seq expression matrix was used to identify differentially expressed genes (DEGs) between HCC and normal liver tissues using the ‘limma’ R package. Unless otherwise indicated, the threshold for DEGs was set at an absolute log2FC > 1 and a false discovery rate < 0.05. These DEGs and polyamine-related genes were intersected to obtain polyamine-related DEGs. Univariate Cox regression was utilized to identify polyamine-related DEGs associated with overall survival.

### 2.5 Consensus clustering based on polyamine-related prognostic genes

According to the univariate Cox regression analysis, the top 12 ranked genes based on hazard ratio were used as features for clustering of HCC patients. Consensus clustering was performed by using the ‘ConsensusClusterPlus’ R package. The optimal number of clusters was determined by cumulative distribution function curve and consensus matrix heatmap.

### 2.6 Functional enrichment analysis

Genes differentially expressed between HCC and normal liver tissues were functionally annotated using the ‘clusterProfiler’ R package by evaluating the enrichment of Gene Ontology (GO) terms, including biological processes, cellular components, and molecular functions. GO terms with an adjusted P value < 0.05 were considered significantly enriched.

### 2.7 Construction and validation of a polyamine-related signature

To establish a polyamine metabolism-based signature, univariate Cox regression analysis was first performed for feature gene selection (univariate *P* < 0.05). The Least Absolute Shrinkage and Selection Operator (LASSO) regression analysis with 10-fold cross-validation was then conducted to eliminate overfitting genes. Subsequently, multivariate Cox regression analysis using the stepwise method (with both forward and backward steps) was performed, and then a polyamine scoring signature termed PAscore was constructed. The PAscore was calculated as the sum of the product of each feature gene’s expression and its corresponding regression coefficient. The TCGA HCC dataset was randomly divided into training and validation cohorts in a 1:1 ratio for model development and validation. Patients were categorized into PAscore-high and PAscore-low groups based on the median PAscore. To assess the independent prognostic significance of PAscore, Cox proportional hazards regression analysis was performed, adjusting for potential confounding factors, including age, gender, tumor stage, and grade. Samples with missing data for any of these clinical covariates were excluded from the analysis.

### 2.8 Immune infiltration analysis

For quantification of immune cell infiltration, three different algorithms were employed: the Estimation of Stromal and Immune cells in Malignant Tumor tissues using Expression data (ESTIMATE) algorithm, the Cell-type Identification by Estimating Relative Subsets of RNA Transcripts (CIBERSORT) algorithm, and single-sample gene set enrichment analysis (ssGSEA).

### 2.9 Prediction of response to immunotherapy and traditional drugs

The Tumor Immune Dysfunction and Exclusion (TIDE) computational method was utilized to predict the response of HCC patients with different PAscores to immune checkpoint inhibitors, including anti-PD-1 and anti-CTLA4. A higher TIDE score indicates reduced responsiveness to immunotherapy (17). Additionally, the stemness score file named “StemnessScores_RNAexp_20170127.2.tsv” was downloaded to analyze cancer stem cell index involved in therapy resistance. The ‘oncoPredict’ R package was employed for drug sensitivity prediction. The necessary training sets were obtained from the Genomics of Drug Sensitivity in Cancer (GDSC) database via oncoPredict’s Open Science Framework (18). Drug sensitivity scores were calculated using the calcPhenotype function.

### 2.10 Cell culture and RNA interference

The human HCC cell lines HepG2 and Huh7 was originally from ATCC and stocked in our laboratory. Cell line authentication was performed before use via short tandem repeats sequencing. Cells were maintained in Dulbecco’s modified Eagle’s medium (DMEM) supplemented with 10% fetal bovine serum, under a humidified atmosphere of 5% CO2 at 37 °C. Small interfering RNA (siRNA) against FIRRE was purchased from GenePharma (Shanghai, China), and sequences were as follows: si-FIRRE-1, 5’-CCAUGUACACCAUCAUCAATT-3’; si-FIRRE-2, 5’- GCCUAGGACCUUUGUG-GUATT-3’.

### 2.11 RNA extraction and quantitative real-time PCR

Total RNA was extracted using RNAiso Plus (TaKaRa) following the manufacturer’s instructions. Complementary DNA (cDNA) was synthesized via reverse transcription with the PrimeScript™ RT reagent Kit (RR047A, TaKaRa). For quantification of gene expression, real-time quantitative PCR was performed on the Roche LightCycler 480 System using TB Green Premix Ex Taq II reagent (RR820A, TaKaRa). Relative gene expression was analyzed by the 2^−ΔΔCT^ formula. Primer sequences for FIRRE were as follows: forward, 5’-CTGTGACCTCGCTTCACTTCT-3’; reverse, 5’- GTGGCAAAGAGCAGAAGATAG-3’.

### 2.12 Cell proliferation and migration assays

HepG2 cells with FIRRE knockdown were seeded into 96-well plates at a density of 2,000 cells per well and incubated overnight. Cell proliferation was evaluated using CCK-8 (Apexbio, USA), measuring absorbance at 450 nm every 24 hours. To evaluate migration ability, 24-well transwell chambers with 8 μm pores (Corning, USA) were utilized. Serum-free medium was added to the upper wells, and medium containing 10% fetal bovine serum was placed in the lower wells. Ten thousand HepG2 cells were seeded in the upper wells and incubated for 48 hours. Migrated cells were then fixed with 4% paraformaldehyde (Beyotime, China) and stained using crystal violet (Beyotime, China).

### 2.13 Statistical analysis

All statistical analyses were performed using R software (version 4.3.1). Group comparisons were conducted using either the two-tailed Student’s t-test or the Wilcoxon test. Survival analysis was carried out using the Kaplan-Meier estimator and the log-rank test. Pearson correlation analysis was used to assess relationships between variables. Differences were considered statistically significant at P < 0.05, unless otherwise indicated.

## 3 Results

### 3.1 Genetic and transcriptional landscape of polyamine-related genes in HCC

**Figure 1:**
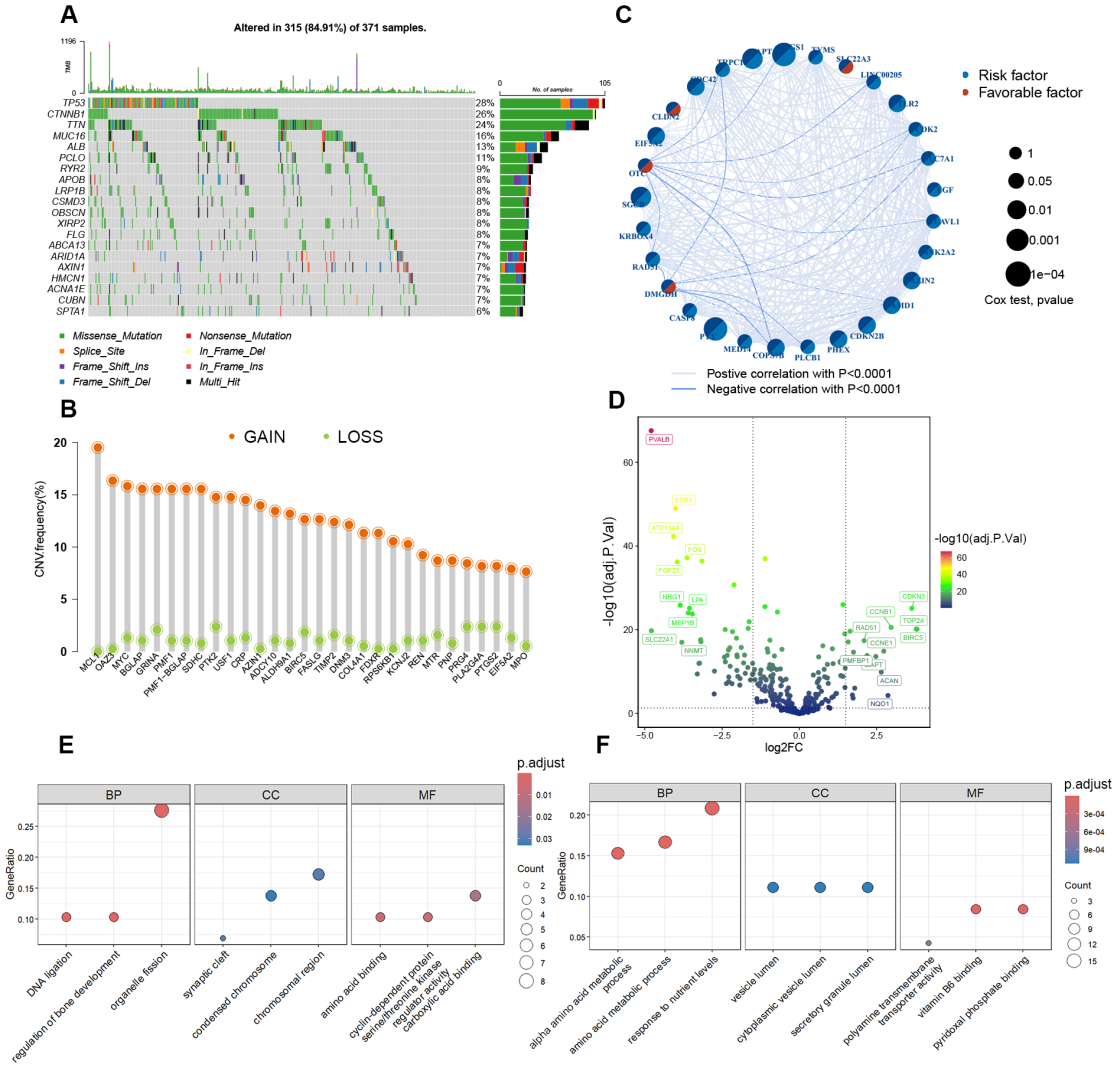
Genetic and transcriptional alterations of polyamine-related genes in HCC. **(A)** Mutation frequency and types of the top 20 most frequently mutated genes in HCC patients from the TCGA cohort. **(B)** The CNV frequency of top 30 polyamine-related genes. The orange circles represent gain-of-function mutations, the blue circles represent loss-of-function mutations. **(C)** Correlation among the polyamine-related genes in HCC. **(D)** Volcano plot showing differentially expressed polyamine-related genes between HCC and normal liver tissues, with normal liver tissues as control. **(E, F)** Gene Ontology (GO) analysis showing the enriched biological processes (BP), cellular components (CC), and molecular functions (MF) of upregulated **(E)** and downregulated **(F)** polyamine-related genes in HCC.

We collected a comprehensive set of 348 polyamine-related genes and analyzed their mutation landscape in HCC patients. Notably, among the top 20 most frequently mutated genes shown in **Figure 1A**, three are polyamine-related genes (*TP53*, *CTNNB1*, *ALB*), each exhibiting a mutation frequency greater than 10%. Copy number variation (CNV) is a common event in polyamine-related genes, with gain CNVs generally occurring at a higher frequency than loss CNVs. For instance, the gain CNV frequency in MCL1 is as high as 20%, while the loss CNV frequency is less than 5% (**Figure 1B**). Furthermore, a polyamine gene network was constructed to depict the comprehensive interactions and associations of polyamine-related genes, as well as their prognostic significance for HCC. Notably, the majority of these polyamine-related genes act as risk factors for the survival of HCC patients (**Figure 1C**).

To gain insights into the role of polyamine metabolism in HCC, we analyzed the expression differences of polyamine-related genes between HCC and normal liver tissues using TCGA cohort. We identified 103 differentially expressed polyamine-related genes in HCC, with 30 genes significantly upregulated and 73 downregulated (**Figure 1D**; **Supplementary Table 2**). Gene Ontology (GO) functional analysis revealed that the upregulated genes are primarily involved in DNA ligation, organelle fission, and protein kinase regulator activity (**Figure 1E**; **Supplementary Table 3**). In contrast, the downregulated genes are associated with amino acid metabolic processes, response to nutrient levels, and pyridoxal phosphate binding (**Figure 1F**; **Supplementary Table 4**). These results indicate an altered polyamine metabolism profile in HCC.

### 3.2 Identification of HCC subtypes based on polyamine metabolism

**Figure 2:**
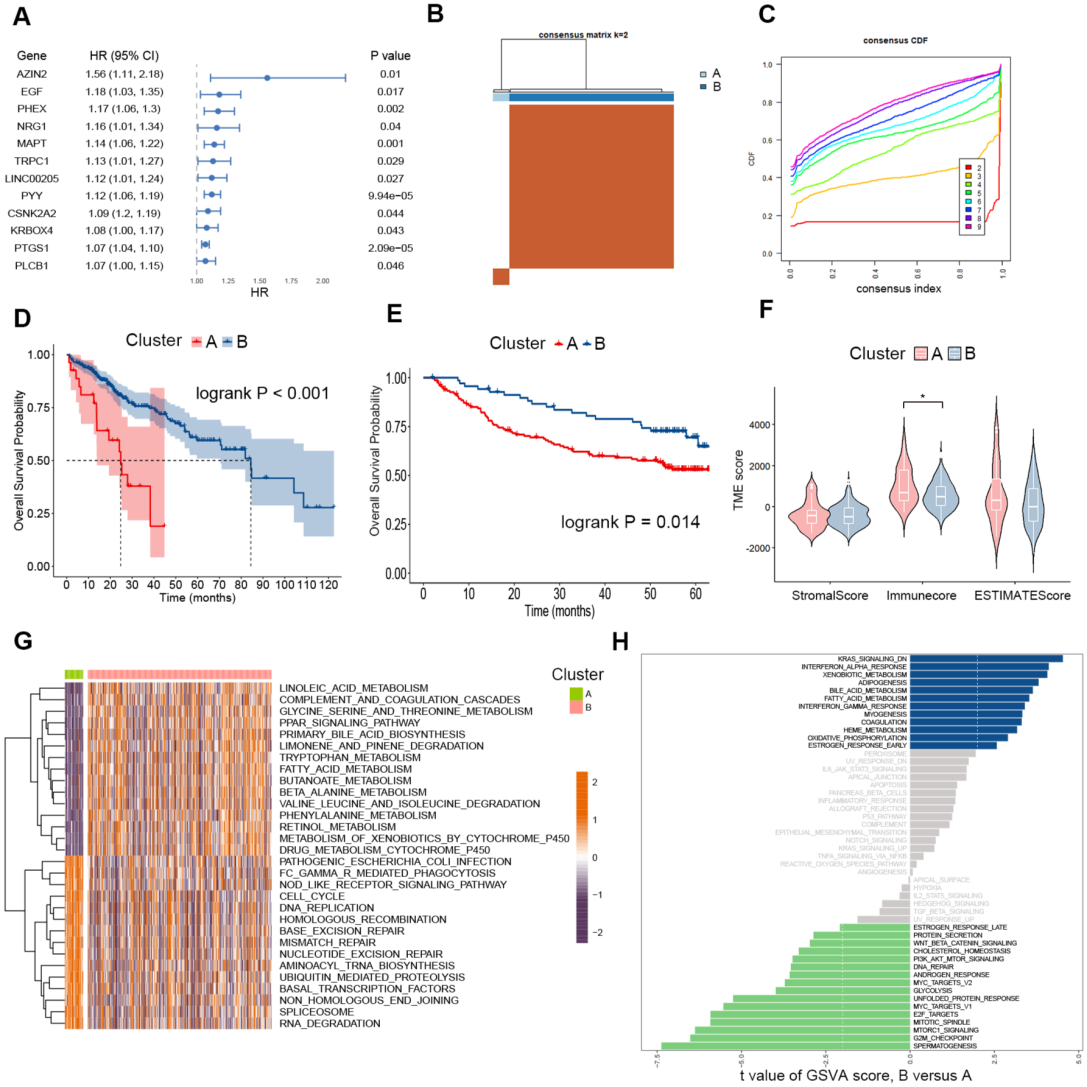
Consensus Clustering in HCC based on specific prognostic polyamine-related genes. **(A)** Forest plot of the top ranked polyamine-related risk genes according to univariate Cox regression analysis. **(B)** Consensus matrix heatmap reflecting the optimal categorization of HCC into two clusters in the TCGA cohort. **(C)** Plot of the cumulative distribution function of the consensus matrix for different k values. **(D)** Kaplan-Meier curves for overall survival in the two clusters of the TCGA cohort. n = 27 for Cluster A, and n = 271 for Cluster **(B, E)** Kaplan-Meier curves for overall survival in the two clusters of the GSE14520 cohort. n = 70 for Cluster A, and n = 151 for Cluster **(B, F)** Violin plots showing the stromal and immune scores of the two clusters of the TCGA cohort. Two-tailed t test was used for two-way comparisons. **(G)** Heatmap displaying the GSVA enrichment analysis results between the two TCGA HCC clusters. **(F)** Differences in pathway activities between the two clusters of the GSE14520 cohort according to the GSVA analysis. *P < 0.05.

To identify the polyamine-related risk genes in HCC, we performed a univariate Cox regression analysis, and the top-ranked genes based on hazard ratios were presented in **Figure 2A**. Given the inter-tumor heterogeneity, we hypothesized that variations in polyamine metabolism activity among patients could, to some extent, account for the differences in prognosis. These top-ranked genes were used as feature genes for consensus clustering, which effectively stratified HCC patients into two distinct clusters (**Figures 2B, C**). The Kaplan–Meier survival analysis revealed significant differences in survival between the two clusters, with Cluster A exhibiting a markedly shorter overall survival time (**Figure 2D**). We next used another HCC cohort to evaluate the utility of these top-ranked polyamine-related risk genes for patient clustering and high-risk patient identification. We next utilized an additional HCC cohort, GSE14520, to assess the effectiveness of these top-ranked polyamine-related risk genes in patient stratification and the identification of high-risk individuals. Consistent with our previous findings, patients were successfully grouped into two clusters (**Supplementary Figures 1A, B**), and Cluster A had a significantly poorer prognosis (**Figure 2E**). These results suggest that the polyamine-related risk genes we selected are potent in identifying high-risk HCC patients.

Next, the ESTIMATE algorithm was employed to gain insights into the differences in the tumor immune microenvironment between the stratified patient groups. The stromal score and ESTIMATE score showed no significant differences between Cluster A and Cluster B, while the immune score was lower in Cluster B (**Figure 2F**). Additionally, GSVA was conducted to assess KEGG pathways activity in each patient within the TCGA cohort. Our analysis revealed that patients in Cluster A exhibited increased pathway activity in cell cycle, DNA replication, and major DNA damage repair mechanisms, including homologous recombination, non-homologous end joining, base excision repair, mismatch repair, and nucleotide excision repair (**Figure 2G**). These biological processes are intimately linked to cancer progression and resistance to chemotherapies. Consistently in the GSE14520 cohort, patients in Cluster B showed enrichment of DNA repair and classical oncogenic signaling pathways, such as glycolysis, PI3K-AKT-mTOR signaling, and WNT-β-catenin signaling. Consistently in the GSE14520 cohort, patients in Cluster B showed enrichment in DNA repair. Classical oncogenic signaling pathways, such as glycolysis, PI3K-AKT-mTOR signaling, and WNT-β-catenin signaling, were also enriched in Cluster B (**Figure 2H**). Taken together, these findings indicate that the polyamine-related risk genes we identified can serve as feature genes for identifying high-risk HCC patients, who exhibit increased oncogenic activities within their tumor niche.

### 3.3 Establishment and validation of a polyamine metabolism-based prognostic signature

**Figure 3:**
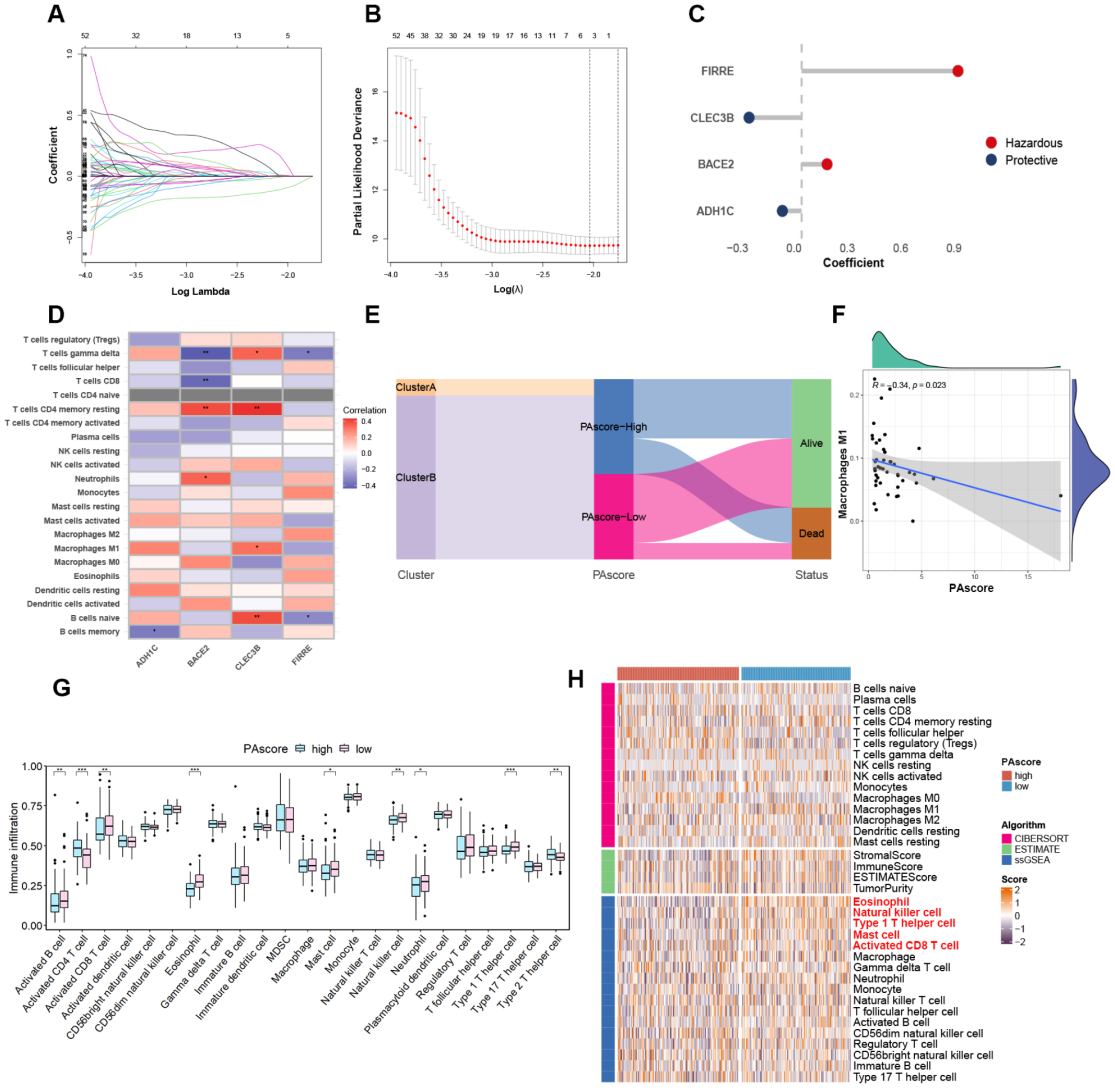
Construction of PAscore prognostic signature. **(A)** LASSO coefficient profiles of the prognostic DEGs in the LASSO-COX regression. **(B)** Cross-validation and the optimal log(λ) value selection of LASSO regression. **(C)** Multivariate Cox coefficients of the four genes in the prognostic signature. **(D)** Heatmap showing the correlation between feature gene expression and immune cell infiltration degrees based on the CIBERSORT algorithm. **(E)** Sankey diagram showing the relationship between clusters, PAscore groups, and survival status in HCC patients of the TCGA cohort. **(F)** Negative correlation of PAscore with M1 macrophage infiltration. **(G)** Boxplot reflecting differences in the infiltration levels of 23 immune cell types within the HCC microenvironment between high- and low-PAscore groups in the TCGA LIHC cohort. Two-tailed t test was used for two-way comparisons. **(H)** Heatmap of tumor-infiltrating immune cell populations in high- and low-PAscore groups, based on CIBERSORT, ESTIMATE, and ssGSEA algorithms. *P < 0.05; **P < 0.01; ***P < 0.001. n = 141 for the low-PAscore group, n = 157 for the high-PAscore group.

Given the impact of polyamine-related genes on oncogenic signaling pathways and the survival of HCC patients, a prognostic model was developed based on the differential expression profiles between patient clusters stratified by polyamine metabolism. For model construction and validation, the TCGA-LIHC cohort was randomly divided into a training set and a test set. We identified 821 DEGs (absolute log2(FC) > 2, adjust P value < 0.05) between the two clusters in the TCGA cohort, of which 165 were found to be correlated with patient survival through univariate Cox regression analysis. These survival-related genes were narrowed down to 5 through LASSO regression analysis (**Figures 3A, B**). Further multivariate Cox regression analysis gave rise to the development of a polyamine-related prognostic signature, termed PAscore, which was derived from the expression of four genes: *FIRRE*, *CLEC3B*, *BACE2*, and *ADH1C*. The weight coefficients for these genes are shown in **Figure 3C**. Genes with a negative coefficient, thereby contributing negatively to the PAscore, are labeled as protective. According to the CIBERSORT algorithm, the expression of the hazardous gene FIRRE is negatively associated with the infiltration of gamma delta T cells (γδ T cells) and naive B cells, while BACE2 expression shows a negative correlation with the infiltration of CD8 T cells and γδ T cells. In contrast, the protective gene CLEC3B is positively associated with the abundance of multiple immune cells, including resting memory CD4 T cells, γδ T cells, M1 macrophages, and naive B cells (**Figure 3D**).

To visualize the relationship between clusters and PAscore subgroups, a Sankey diagram was used, showing that all patients in Cluster A were classified into the high PAscore group (**Figure 3E**). PAscore is negatively correlated with infiltrated M1-polarized macrophages (**Figure 3F**), which possess tumor-killing effects, in contrast to M2-polarized macrophages that promote polyamine production and support tumor growth (19). Furthermore, a comparison between the high PAscore group and the low PAscore group revealed that the infiltration levels of activated B cells, activated CD8 T cells, neutrophils, natural killer cells, and type 1 T helper cells were all significantly lower in the high PAscore group (**Figures 3G, H**). Consistent with the results observed in the TCGA LIHC dataset, high-PAscore patients in the LICA-FR dataset also exhibited reduced infiltration of activated CD8 T cells, eosinophils, and type 1 T helper cells (**Supplementary Figure 2**). These results indicate that the tumor microenvironment in the high PAscore group exhibits lower immune activity.

### 3.4 The association of PAscore with patient prognosis and clinicopathologic characteristics

**Figure 4:**
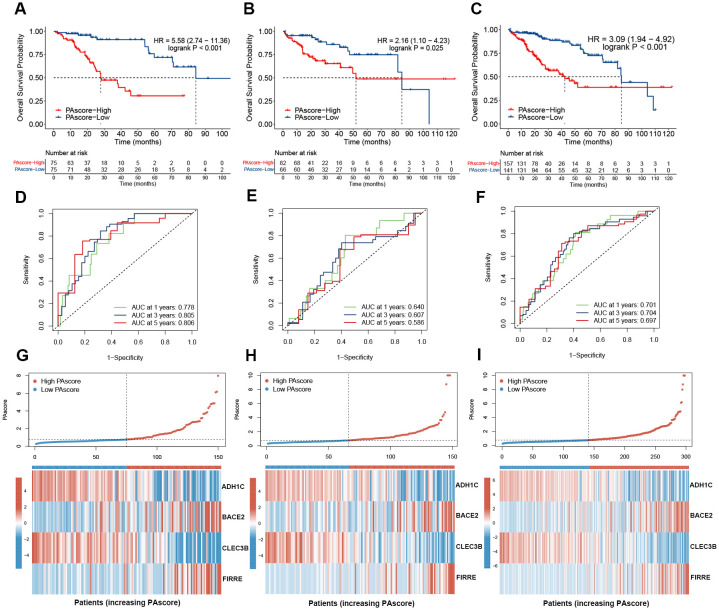
High PAscore indicates poor prognosis. **(A–C)** Kaplan-Meier curves for overall survival in TCGA HCC patients with high- and low-PAscore in the training **(A)**, test **(B)**, and combined **(C)** sets. **(D–F)** Time-dependent receiver operating characteristic curves of the PAscore signature for predicting 1-, 3-, and 5-year survival of HCC patients across the training **(D)**, test **(E)**, and combined **(F)** sets. **(G-I)** PAscore distribution and expression heatmap of the four signature genes in high and low PAscore groups across the training **(G)**, test **(H)**, and combined **(I)** sets.

Kaplan-Meier survival analysis in the training set revealed that patients with higher PAscore had significantly worse overall survival, and similar results were observed in the test set (**Figures 4A, B**). When the training and test sets were combined for analysis, a high PA score effectively predicts poor prognosis (HR = 3.09, logrank P < 0.001) (**Figure 4C**). The ROC curves and their AUC values for 1-year, 3-year, and 5-year survival in the training, test, and combined sets are presented in **Figures 4D–F**. The AUC values for 1-, 3-, and 5-year survival were 0.778, 0.805, and 0.806 in the training set, and 0.640, 0.607, and 0.586 in the test set, respectively. These results demonstrate the reliable predictive ability of our prognostic signature. In addition, the expression patterns of the four genes used in the prognostic signature were shown in the heatmaps (**Figures 4G–I**). Patients in the high PAscore group exhibited increased expression of the hazardous genes FIRRE and BACE2, but lower expression of the protective genes ADH1C and CLEC3B.

**Figure 5:**
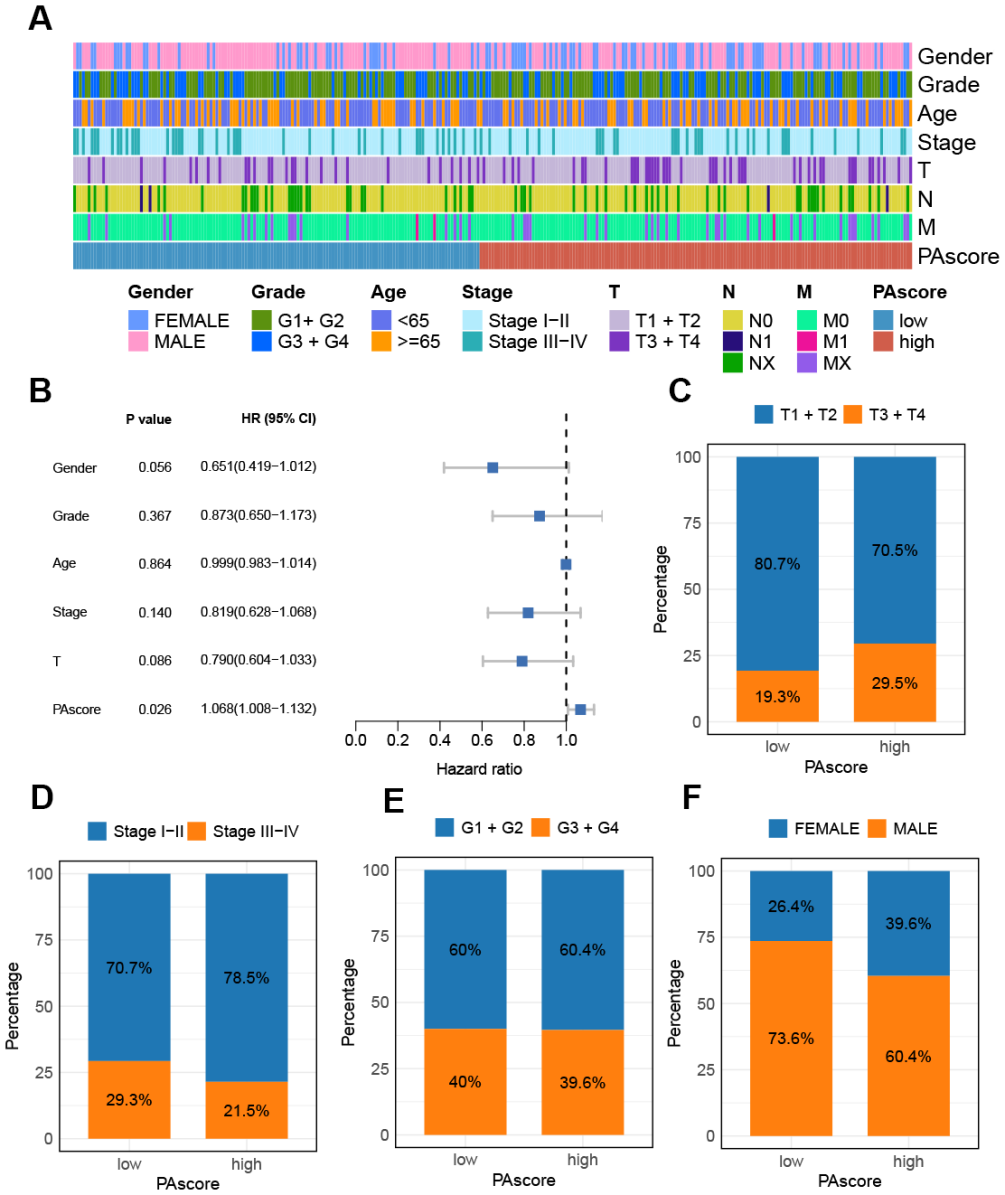
Relationship between PAscore and the clinicopathologic features of HCC patients. **(A)** A band chart of PAscore and clinical features of HCC patients. **(B)** Forest plot showing prognostic significance of age, gender, tumor stage, T and PAscore based on Cox regression analysis. **(C)** The proportion of patients with different T classifications (T1 + T2 vs. T3 + T4) in the high- and low-PAscore groups. **(D)** The proportion of male and female patients in the high- and low-PAscore groups. **(E)** The distribution of patients with distinct tumor grades in different PAscore groups. **(F)** The distribution of patients with distinct tumor stages in different PAscore groups.

The relationship between PAscore and the clinicopathologic features of HCC patients was then evaluated. The tumor stage, grade, and TNM classification for patients stratified by PAscore were presented in **Figure 5A**. Among the clinicopathologic characteristics analyzed, PAscore emerged as an independent factor associated with poor prognosis (**Figure 5B**). Furthermore, the proportion of patients with a T3 or T4 pathologic stage was significantly higher in the high PAscore group compared to the low PAscore group, while the proportion of patients with stage III-IV was slightly lower in the high PAscore group (**Figures 5C, D**). There was no significant difference in the proportion of patients with high tumor grades between the two PAscore groups, and fewer male patients were in the high PAscore group (**Figures 5E, F**).

### 3.5 Heterogeneity in polyamine metabolism within the tumor cells of the HCC microenvironment

**Figure 6:**
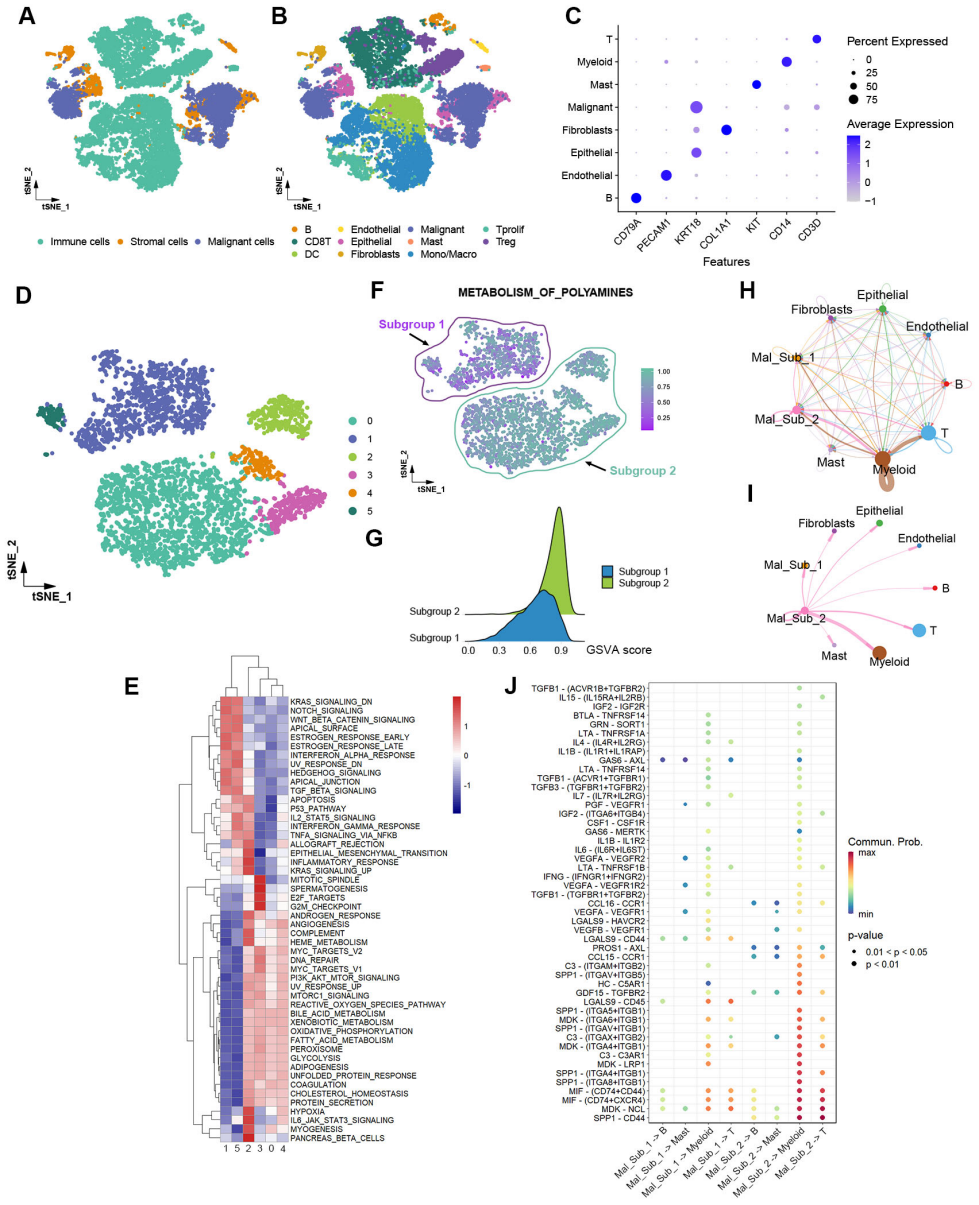
Single-cell RNA sequencing analysis reveals the heterogeneity of polyamine metabolism in HCC cells and varying interactions with tumor-infiltrating immune cells. **(A)** t-SNE plot showing immune cells, stromal cells, and malignant cells within the HCC microenvironment. **(B)** t-SNE plot of the eleven cell types. **(C)** Dot plot of the expression levels of marker genes in the major cell types. **(D)** t-SNE plot of 4,205 malignant cells grouped into six distinct clusters. **(E)** Heatmap of the GSVA scores of the hallmark pathway gene signatures in the six clusters of malignant cells. **(F)** t-SNE plot color-coded for polyamine metabolic activity in different malignant cell clusters that are categorized into two subgroups based on polyamine metabolic activity levels. **(G)** Ridge plot showing the distribution of GSVA scores for polyamine metabolism in Subgroup 1 and Subgroup 2. **(H)** Overall ligand-receptor-based communication strength among different cell types. **(I)** Ligand-receptor communications from Subgroup 2 malignant cells to other cell types. **(J)** Signal identification through comparative analysis of ligand-receptor pair-mediated communication probabilities between malignant cell subgroups and non-malignant cell types. Mal_Sub_1, Subgroup 1 malignant cells; Mal_Sub_2, Subgroup 2 malignant cells.

Intratumor heterogeneity, an essential property of cancers, is crucial to malignant phenotypes and significantly influences treatment response (20). We analyzed the polyamine metabolic activity of malignant cells using single-cell RNA sequencing data from two HCC patients. A total of 22,330 cells, including immune cells, malignant cells, and stromal cells, were annotated into eleven distinct cell types that represent the composition of the HCC ecosystem, consisting of fibroblasts, endothelial cells, epithelial cells, malignant cells, macrophages, monocytes, DCs, mast cells, B cells, T cells (**Figures 6A, B**). Specific markers for each cell type were shown in **Figure 6C**. Reclustering the 4,205 malignant cells revealed 6 clusters (**Figure 6D**). GSVA analysis of the hallmark pathway gene signatures revealed that these 6 clusters could be further clustered into two subgroups. Remarkably, Subgroup 2 (Clusters 0, 2, 3, 4) exhibited significantly higher polyamine metabolism activity compared to Subgroup 1 (Clusters 1, 5) (**Figures 6E–G**). Furthermore, cells in Subgroup 2 exhibited increased activity in oxidative phosphorylation, glycolysis, DNA repair, angiogenesis, and MYC targets, while showing reduced activity in the interferon alpha response, apoptosis, and p53 pathway (**Figure 6E**). These findings underscore the polyamine metabolic heterogeneity among malignant cells and the positive correlation between polyamine metabolic activity and oncogenic signaling pathway activity at the single-cell level.

Ligand-receptor-mediated intercellular communication landscape was shown in **Figure 6H**. Compared to Subgroup 1, Subgroup 2 cells showed increased crosstalk with myeloid cells. Notably, the communication between Subgroup 2 cells and myeloid cells was stronger than that with other cell types (**Figures 6I, J**). The PROS1-AXL, EDA-EDA2R, C3-(ITGAX+ITGB2), IL17A-(IL17RA+IL17RC), TGFA-EGFR, and NPPC-NPR2 ligand-receptor interactions primarily mediate the increased crosstalk between Subgroup 2 cells and myeloid cells. Meanwhile, MDK-NCL, MIF-(CD74+CXCR4), and NPPC-NPR2 contribute to the enhanced communication between Subgroup 2 cells and T cells (**Supplementary Figure 3**). These distinct ligand-receptor interaction profiles may underlie the varying immune evasion capacities of Subgroup 1 and Subgroup 2 cells. For example, the PROS1-AXL pathway mediates the interaction between MICA^+^ tumor cells and MMP9^+^ macrophages, to facilitate tumor immune escape in advanced HCC (21). The MDK-NCL signal is associated with suppressed immune activity in endometrial carcinoma (22). Further investigation is needed to determine whether the communication between tumor cells and myeloid cells affects polyamine metabolism and malignant phenotypes in tumor cells.

### 3.6 Higher PAscore is associated with reduced sensitivity to immunotherapy and other antitumor drugs

**Figure 7:**
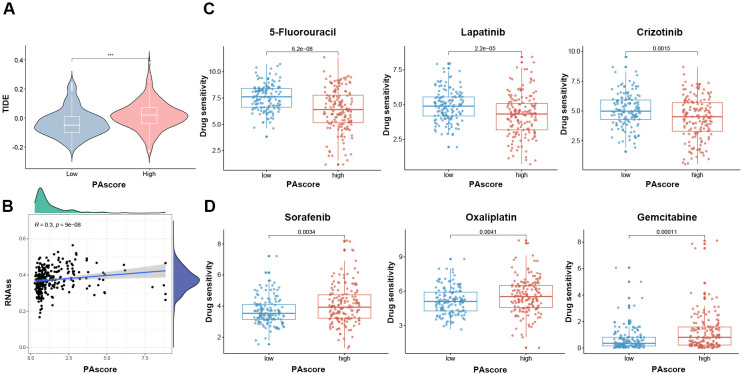
PAscore signature is positively associated with reduced sensitivity to immunotherapy in HCC. **(A)** Comparison of TIDE scores between high- and low-PAscore patients. **(B)** Correlation between PAscore and cancer stem cell index. **(C)** Reduced sensitivity to 5-fluorouracil, lapatinib, and crizotinib in patients with higher PAscore. **(D)** Increased sensitivity to sorafenib, oxaliplatin, and gemcitabine in patients with higher PAscore. ***, *P* < 0.001. **(A, C, D)** Two-tailed t test was used for two-way comparisons.

To explore the clinical application of our polyamine-related signature in predicting drug response, we analyzed the relationship between the PAscore and sensitivity to immune checkpoint inhibitors and other commonly used antitumor drugs. Our analysis revealed that patients with higher PAscores exhibited elevated TIDE scores (**Figure 7A**), suggesting that immune checkpoint inhibitors are likely less effective in these individuals. Accumulating evidence demonstrates that cancer stem cells contribute to therapy resistance (23, 24), and a significant positive association (R = 0.3, P < 0.001) was observed between the PAscore and CSC index (**Figure 7B**). We utilized the oncoPredict package to estimate the differences in drug sensitivity between high and low PAscore groups. Patients with a higher PAscore exhibited greater resistance to drugs such as 5-fluorouracil, lapatinib, and crizotinib (**Figure 7C**). However, a higher PAscore was associated with increased sensitivity to sorafenib, oxaliplatin, and gemcitabine (**Figure 7D**), suggesting that patients with a higher PAscore might derive greater benefit from these drugs. These results suggest that the polyamine-related signature has the potential to predict response to immunotherapy and other antitumor drugs in HCC patients.

### 3.7 Knockdown of FIRRE, a constitutive gene of polyamine-related signature, impairs HCC cell proliferation and migration

**Figure 8:**
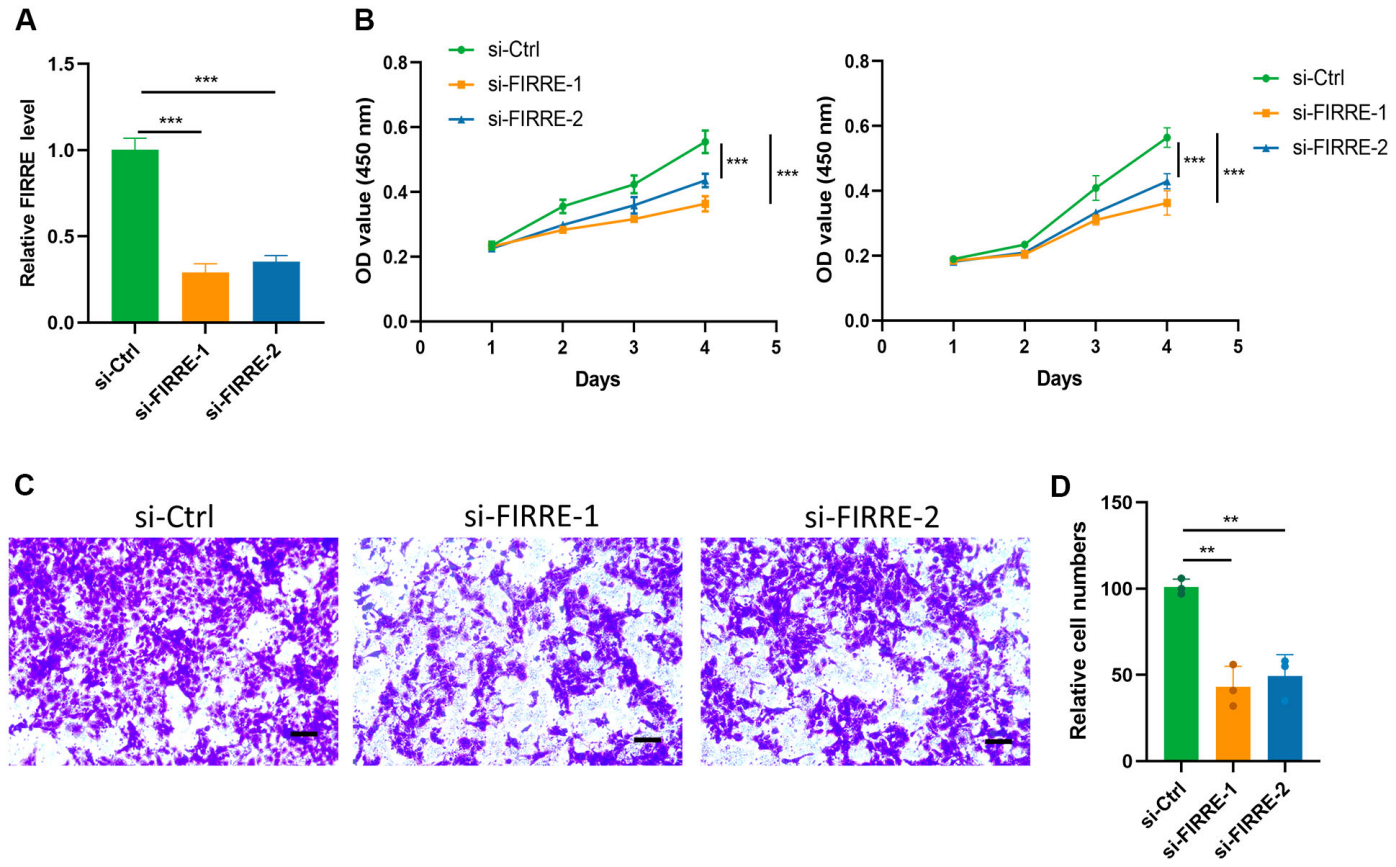
Polyamine-related signature impairs HCC cell proliferation and migration. **(A)** qRT-PCR analysis of FIRRE expression, a key gene in the polyamine-related signature, in HepG2 cells following siRNA transfection. **(B)** CCK8 assay assessing the proliferation capacity of HepG2 (left) and Huh7 (right) cells after FIRRE knockdown. **(C)** Transwell assay evaluating the migration ability of HepG2 cells after FIRRE knockdown. **(D)** Relative quantification of the number of migrated cells in the transwell assay. **P < 0.01, ***P < 0.001, scale bar, 200 µm. Two-tailed t test was used for two-way comparisons.

Given the significant influence of FIRRE on the PAscore, as indicated by its high coefficient within the PAscore signature, FIRRE was selected for further analysis. Knockdown of FIRRE was achieved using small interfering RNA (**Figure 8A**). The CCK8 assay demonstrated that FIRRE knockdown significantly reduced the proliferation of HepG2 cells (**Figure 8B**). Additionally, silencing FIRRE markedly diminished the migration capacity of HCC cells, as revealed by the transwell assay (**Figures 8C, D**). These findings suggest that polyamine-related signature plays a critical role in the regulation of HCC cell proliferation and migration.

## 4 Discussion

Dysregulated polyamine metabolism is common in cancers, increased intracellular polyamine pools contribute to tumor proliferation and immune evasion (14, 25). Therefore, targeting polyamine metabolism has been identified as a promising therapeutic strategy. Studies have increasingly highlighted the significant role of polyamines in the progression of HCC. For instance, spermine has been shown to promote HCC progression by establishing immune privilege, achieved through the maintenance of N-glycosylation and stability of PD-L1 (26). Hitherto in HCC, the effect of polyamine metabolism on regulating the immune landscape within the tumor microenvironment and its influence on the response to immunotherapy have remained largely unknown, and the predictive significance of polyamine metabolic activity in patients’ prognosis requires further assessment.

In this study, we identified polyamine-related genes correlated with overall survival in HCC patients. Based on the top-ranked polyamine-related prognostic genes, HCC patients could be categorized into two distinct clusters, each differing in specific cellular signaling activity. Cluster A was characterized by a worse prognosis and elevated activities in DNA damage repair, DNA replication, and cell cycle processes. These findings were corroborated by analyses of an independent HCC cohort, where Cluster A also exhibited shorter overall survival and heightened DNA repair capacities. The rapid proliferation of tumor cells relies on efficient DNA replication and fluent cell cycle progression, which are, in part, dependent on polyamine abundance (27, 28). Studies have revealed the biological function of polyamines in protecting DNA from single-strand breaks and promoting homology-directed repair of DNA double-strand break (29, 30). In HCC patients, enhanced DNA repair capability is correlated with worse survival (31). Consistent with these findings, our results here further underscore the involvement of polyamines in single- and double-strand DNA break repair. Oncogenic signaling pathways, such as Wnt/β-catenin and PI3K/AKT/mTOR, were enriched in Cluster A, supporting the notion of crosstalk between polyamine metabolism and oncogenic signaling (32).

Based on DEGs between Cluster A and Cluster B, we developed a polyamine-related signature, termed PAscore, comprising four genes: *FIRRE*, *CLEC3B*, *BACE2*, and *ADH1C*. The PAscore signature effectively serves as a prognostic marker that indicates poor overall survival and reduced responsiveness to immune checkpoint blockade. It also reflects lower infiltration of specific immune cell types, including activated CD8 T cells, natural killer cells, activated B cells, and neutrophils. The reduced presence of tumor-infiltrating lymphocytes aligns with previous findings that polyamines are involved in suppressing clonal deletion of B cells and that optimal induction of cytolytic T lymphocytes depends on elevated polyamine levels (33, 34).

Among the four genes in the PAscore signature, the long non-coding RNA FIRRE has the highest coefficient, indicating its significant impact on the PAscore. Like ornithine decarboxylase (ODC), the rate-limiting enzyme in polyamine biosynthesis, FIRRE is also transcriptionally activated by MYC (10, 35). Although there is no direct evidence indicating that FIRRE regulates polyamine metabolism, inhibition of tumor glycolysis has been shown to reduce both ODC expression and polyamine levels (36), and FIRRE has been found to enhance glycolytic activity by promoting the transcription of the glycolytic enzyme PFKFB4 (37). These findings suggest that FIRRE may indirectly influence polyamine metabolism. Additionally, FIRRE has been shown to regulate the expression of immunomodulatory genes, such as VCAM1 and TNF-α (38), indicating its role in modulating the tumor immune microenvironment. Our *in vitro* experiments showed that FIRRE knockdown significantly inhibited the proliferation and metastasis of HCC cells, corroborating the previously reported pro-tumor effects of FIRRE (37, 39). BACE2 is a β-secretase protein that is overexpressed in cancers. In ocular melanoma, BACE2 has been found to mediate intracellular calcium release from the endoplasmic reticulum and support tumor progression by regulating the expression of TMEM38B, a cation channel protein in the endoplasmic reticulum membrane (40). Our prognostic signature indicates that BACE2 has an adverse effect on the prognosis of HCC patients. Notably, intracellular calcium content regulates polyamine transport (41), suggesting that BACE2 may be involved in polyamine metabolism, although further experimental investigations are needed. CLEC3B and ADH1C are indicators of a better prognosis for HCC patients according to the PAscore signature, which is consistent with previous studies (42, 43). CLEC3B is a secreted protein that can suppress angiogenesis through exosome-mediated inhibition of VEGF (44).

HCC is characterized by metabolic heterogeneity, which can be used to stratify patients (45). Our analysis of single-cell RNA sequencing data of HCC revealed polyamine metabolic heterogeneity among malignant cells, which could be categorized into two subgroups based on polyamine metabolic activity, with each subgroup exhibiting distinct hallmark pathway activities. For instance, the subgroup with increased polyamine metabolic flux showed enhanced activity in angiogenesis, DNA repair, MYC targets, PI3K/AKT/mTOR signaling, and glycolysis. These single-cell-based analyses of crosstalk between polyamine metabolism and oncogenic signaling are supported by previous research (12, 30, 46–48). High polyamine pools in tumors trigger an immunosuppressive microenvironment. Studies in melanoma and breast cancer mouse models revealed that reduced tumor polyamine abundance partially mitigated immunosuppression by decreasing the survival of tumor-associated myeloid cells (49). Our analysis indicated increased communication between HCC cells with high polyamine metabolic activity and myeloid cells, which reflects a similar scenario within the HCC environment where polyamines facilitate the interaction between malignant cells and myeloid cells to contribute to the development of a tumor-permissive niche.

Polyamines function as crucial regulators not only in fundamental cellular metabolism but also in immune regulation within the tumor microenvironment. Polyamines influence the differentiation of CD4^+^ T cells and the cytotoxic function of CD8^+^ T cells. For example, in glioblastoma, cancer cell-derived spermidine has been shown to decrease the tumor-infiltrating number of CD8^+^ T cells and impair their cytotoxic activity through altering their cytokine profile (50). Tumor-derived polyamines also favor M2 macrophages to support tumor growth, paracrine secretion of polyamines by M2 macrophages has been found to suppress the activity of T cells and dendritic cells in the tumor microenvironment (51, 52). Targeting polyamine synthesis with difluoromethylornithine (DFMO), an irreversible ODC inhibitor, has demonstrated significant improvements in overall survival in high-risk neuroblastoma patients (53). In addition to DFMO, AMXT-1501 dicaprate, a polyamine transport inhibitor that prevents uptake of extracellular polyamines, is also being investigated as a therapeutic approach targeting the polyamine pathway. A clinical trial has been initiated to assess the intra-tumoral extracellular metabolic impact of DFMO and AMXT 1501 in patients with diffuse or high-grade glioma (NCT05717153). These collectively highlight the potential of polyamine-blocking therapies as a promising therapeutic strategy for cancer treatment.

There are certain limitations in our study. Firstly, while the prognostic significance of the polyamine-related signature was validated, further validation of its predictive value for survival and immunotherapy efficacy in a larger HCC cohort would enhance the reliability of this signature. Secondly, while we identified four signature genes and experimentally investigated the role of FIRRE in regulating the proliferation and migration of HCC cells, further research is necessary to elucidate the potential functions and underlying mechanisms of these four genes in HCC progression. Lastly, although our study revealed increased crosstalk between HCC cells with enhanced polyamine metabolic activity and myeloid cells, additional studies are required to uncover the molecular mechanisms driving this crosstalk and its consequences.

In summary, we present a polyamine-related signature that predicts prognosis, immune landscape, and immunotherapy response in HCC. We highlight polyamine metabolic heterogeneity among HCC cells, which is associated with distinct hallmark pathway signatures. Additionally, we demonstrate that, compared to other cell types within the HCC microenvironment, communication between malignant cells with high polyamine metabolic activity and myeloid cells is more active. Targeting polyamine metabolism and this enhanced crosstalk may offer more effective combination immunotherapy strategies.

## Data Availability

The raw data supporting the conclusions of this article will be made available by the authors, without undue reservation.

## Supplementary material

Supplementary Figure 1 Consensus Clustering in HCC GSE14520 dataset based on specific prognostic polyamine-related genes. **(A)** Consensus matrix heatmap reflecting the optimal categorization of HCC into two clusters in GSE14520 cohort. **(B)** Plot of the cumulative distribution function of the consensus matrix for different k values in GSE14520 cohort.

Supplementary Figure 2 Boxplot illustrating the differences in the infiltration levels of 23 immune cell types within the HCC microenvironment between the high- and low-PAscore groups in the ICGC LICA-FR cohort. *, *P* < 0.05; **, *P* < 0.01; ***, *P* < 0.001. n = 80 for both groups. Two-tailed t test was used for two-way comparisons.

Supplementary Figure 3 Ligand-receptor interactions that mediate the distinct crosstalk between malignant cells (Subgroup 1 and Subgroup 2) and other cell types within the tumor microenvironment.
