# Supplementary figures and images for: Heterogeneity in polyamine metabolism dictates prognosis and immune checkpoint blockade response in hepatocellular carcinoma
Source: Front Immunol. 2025 Feb 6;16:1516332. doi: 10.3389/fimmu.2025.1516332 (PMC11839726; doi:10.3389/fimmu.2025.1516332)

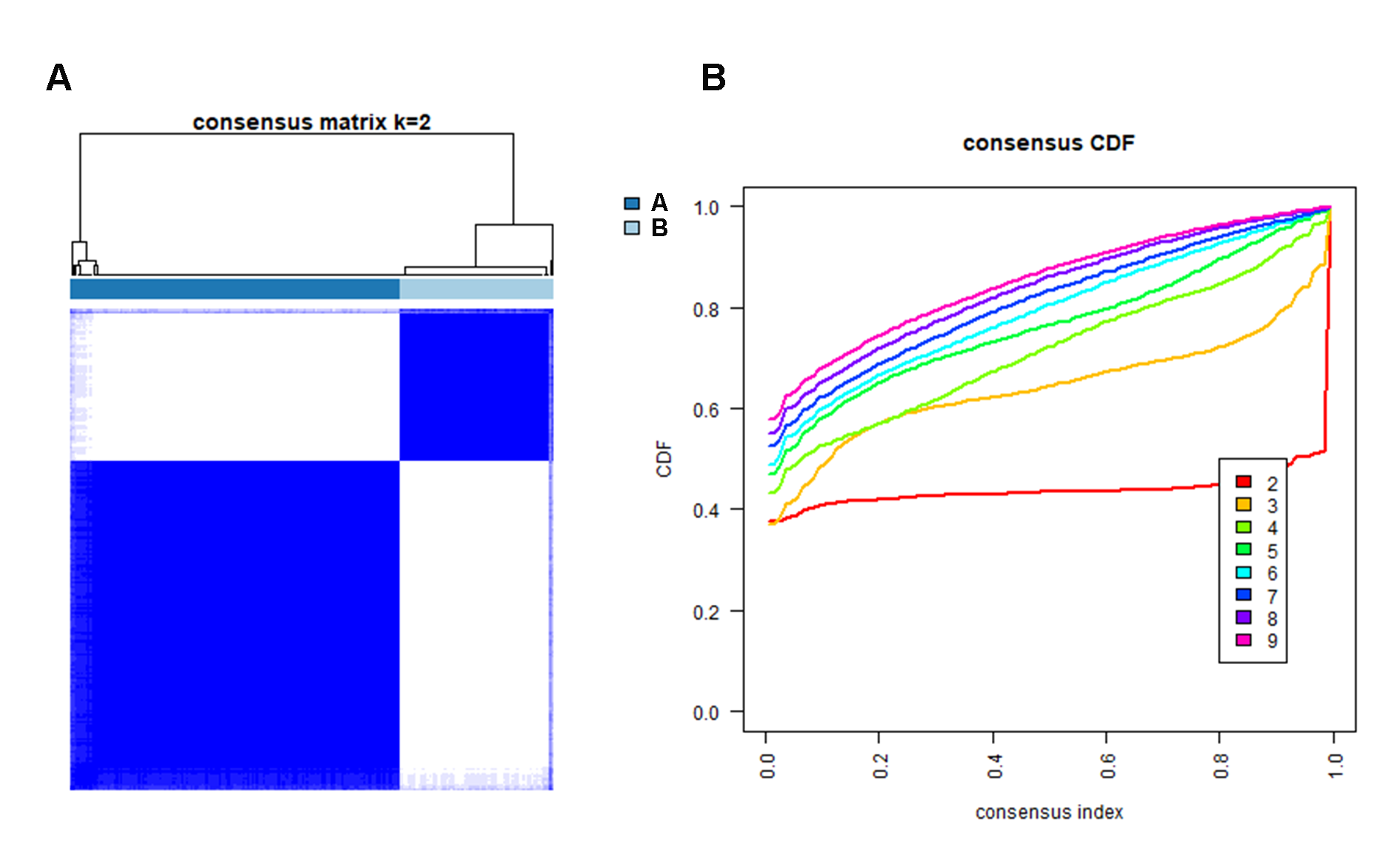

Supplement: Supplementary Figure 1 — Consensus Clustering in HCC GSE14520 dataset based on specific prognostic polyamine-related genes. (A) Consensus matrix heatmap reflecting the optimal categorization of HCC into two clusters in GSE14520 cohort. (B) Plot of the cumulative distribution function of the consensus matrix for different k values in GSE14520 cohort. [file Image1.tif]

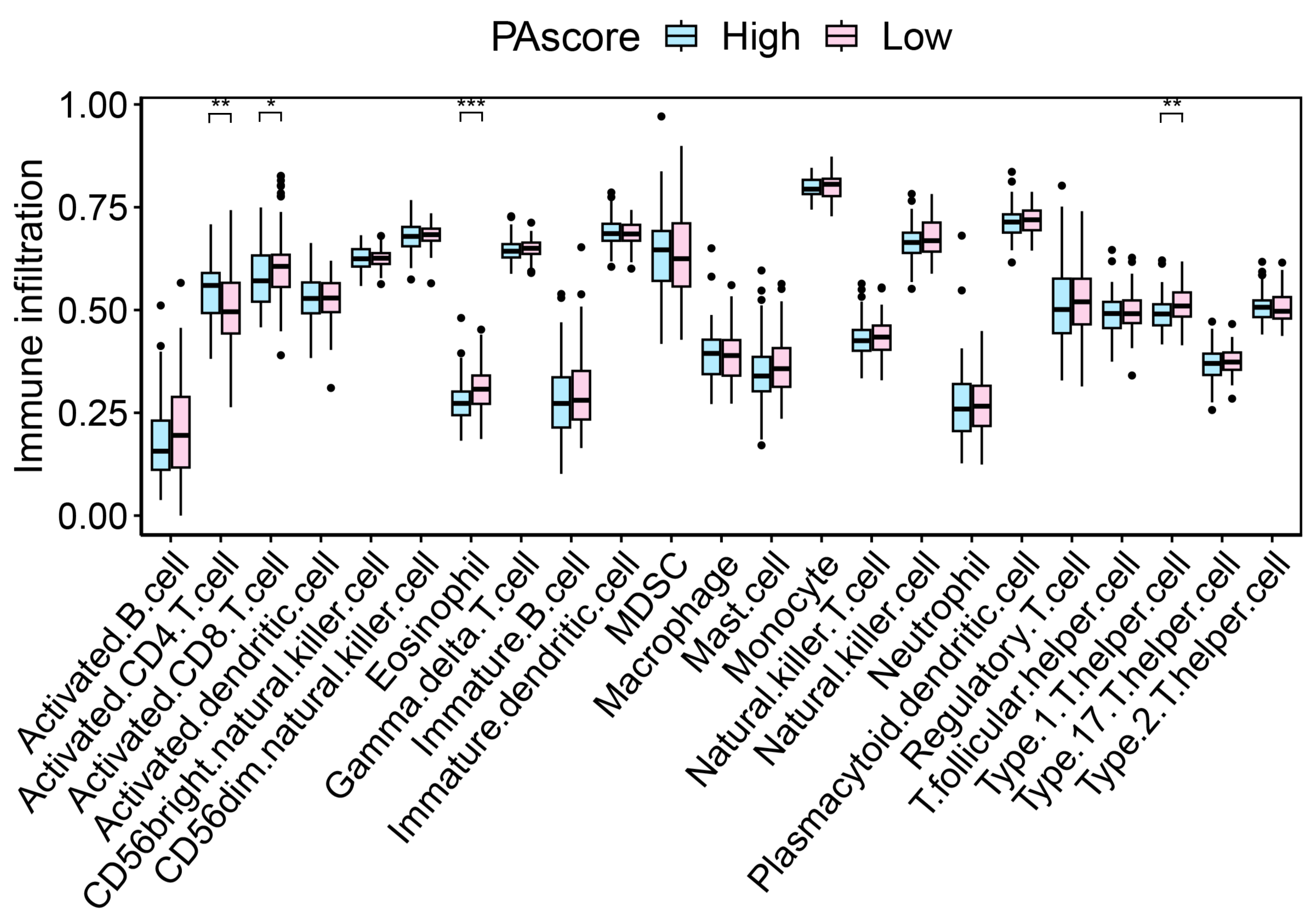

Supplement: Supplementary Figure 2 — Boxplot illustrating the differences in the infiltration levels of 23 immune cell types within the HCC microenvironment between the high- and low-PAscore groups in the ICGC LICA-FR cohort. *, P < 0.05; **, P < 0.01; ***, P < 0.001. n = 80 for both groups. Two-tailed t test was used for two-way comparisons. [file Image2.png]

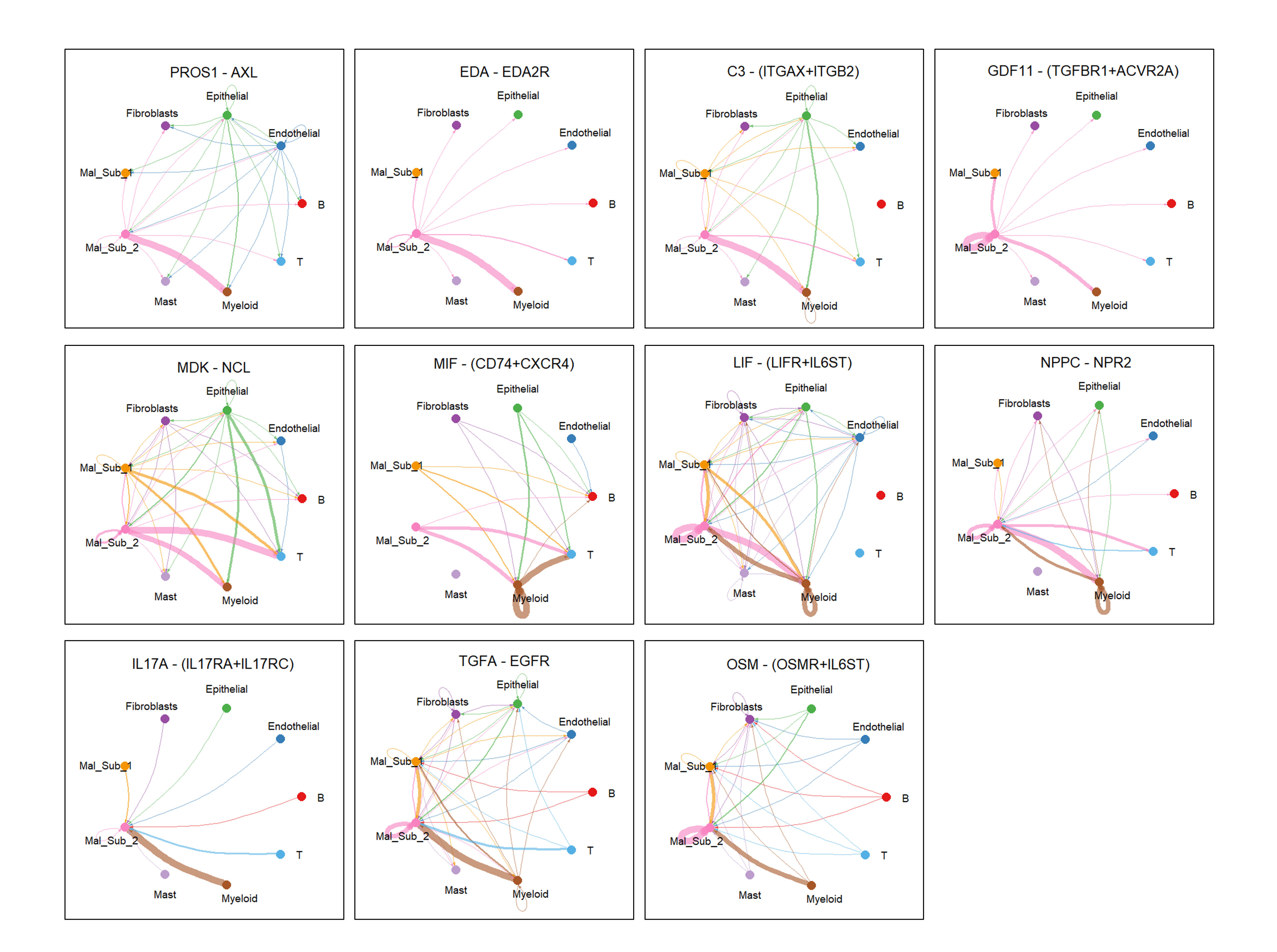

Supplement: Supplementary Figure 3 — Ligand-receptor interactions that mediate the distinct crosstalk between malignant cells (Subgroup 1 and Subgroup 2) and other cell types within the tumor microenvironment. [file Image3.png]
